# Dose Adjustment of Intravenous Immunoglobulin Therapy With Serum IgG Monitoring to Prevent Hyperviscosity in a 13-Year-Old Boy With Kawasaki Disease

**DOI:** 10.7759/cureus.80317

**Published:** 2025-03-09

**Authors:** Shin Takeo, Shoichiro Kanda, Keiichi Takizawa, Yuko Kajiho, Akiko Kinumaki

**Affiliations:** 1 Pediatrics, The University of Tokyo, Tokyo, JPN

**Keywords:** adolescent medicine, individualisation of drug dosage, intravenous immunoglobulin (ivig), kawasaki disease (kd), retropharyngeal edema

## Abstract

Kawasaki disease (KD) primarily affects infants and young children, with cases in patients over 10 years of age being relatively rare. The standard treatment involves intravenous administration of high-dose intravenous immunoglobulin (IVIG) at 2 g/kg. In pediatric medicine, drug dosages are typically adjusted according to body size; however, in older children, body size-based dosing often results in excessively high doses, and adult dosages are frequently used as upper limits. Notably, no such upper limit exists for IVIG. Given that IVIG administration can cause severe cardiac and neurological complications due to hyperviscosity syndrome, careful dose management is essential.

Here, we report the case of a 13-year-old KD patient weighing 53 kg who underwent repeated high-dose IVIG therapy. Serum IgG levels, total protein concentration, and hematocrit were monitored over time as an indicator of blood viscosity, allowing for dose adjustments. As a result, the patient successfully completed the treatment course without experiencing major adverse effects. Notably, the adjusted IVIG regimen effectively prevented the development of hyperviscosity syndrome. Furthermore, the patient did not develop coronary artery aneurysms, indicating that the treatment maintained both safety and therapeutic efficacy in managing Kawasaki disease. On the other hand, the increase in serum IgG levels following IVIG at a dose of 2 g/kg was greater in this patient compared to younger children receiving the same treatment.

This case highlights the necessity of monitoring IgG levels to balance the efficacy and safety of IVIG therapy in older KD patients. However, there is no established threshold for IgG levels that would indicate a safe upper limit for IVIG administration, highlighting the need for further investigation.

## Introduction

Kawasaki disease (KD) is an idiopathic vasculitis primarily affecting infants and young children. It is characterized by fever, bilateral conjunctival injection, erythema of the lips and strawberry tongue, skin rash, changes in the extremities, and non-suppurative cervical lymphadenopathy [1, 2]. If a patient exhibits five or more of these six clinical features, a diagnosis of KD is established, whereas the presence of four features meets the criteria for incomplete KD. International guidelines recommend intravenous immunoglobulin (IVIG) at 2 g/kg as the first-line treatment [3]. Intravenous immunoglobulin demonstrates efficacy in approximately 80% to 90% of KD cases, effectively reducing the risk of coronary artery aneurysms to below 5% with appropriate treatment. However, 10% to 20% of patients exhibit IVIG resistance, necessitating additional therapy. Its mechanisms include proinflammatory cytokine suppression, Fc receptor blockade, and regulatory T cell activation, collectively mitigating acute inflammation and preventing coronary artery complications.

Intravenous immunoglobulin is known to cause various adverse effects, most commonly mild reactions such as fever and headache [4]. However, in rare cases, it may lead to severe complications, including hyperviscosity syndrome.

Hyperviscosity syndrome is a pathological condition characterized by increased blood viscosity, resulting in impaired microcirculation and subsequent ocular, neurological, and cardiovascular complications. Intravenous immunoglobulin administration may precipitate hyperviscosity due to the rapid elevation of serum IgG levels, leading to a marked increase in plasma protein concentration and blood viscosity [5].

In pediatric patients, drug dosages are typically adjusted based on body size, with an upper limit often set at the adult dose. However, IVIG for KD lacks a well-established upper limit, leading to proportionally increased dosages in larger children. Consequently, in children with greater body size, the standard 2 g/kg IVIG regimen may result in excessive elevations in serum IgG levels, raising concerns about the potential for various adverse effects. Evidence regarding the safety of high-dose IVIG in older pediatric patients is limited.

This report describes a case of KD in an older child where serum IgG monitoring was used to adjust IVIG dosing, aiming to balance treatment efficacy with the risk of hyperviscosity-related complications.

## Case presentation

A previously healthy 13-year-old boy, 158 cm tall and weighing 53 kg, presented with fever and painful bilateral cervical swelling. His past medical history included bronchial asthma, constipation, and inguinal hernia, with no known allergies. His medications included a salmeterol/fluticasone inhaler, montelukast, and a polyethylene glycol formulation.

Two days before admission, on illness day 2, he developed a fever and neck pain. On admission, on illness day 4, a primary care physician noted marked cervical swelling, leukocytosis, and elevated C-reactive protein (CRP) levels, raising suspicion for a retropharyngeal abscess and prompting referral to our hospital. On physical examination, his body temperature was 38.3°C, heart rate was 90 beats per minute, blood pressure was 113/63 mmHg, respiratory rate was 24 breaths per minute, and oxygen saturation was 99% on room air. A mild left conjunctival injection was noted. His lips were dry and cracked, though without erythema or swelling. Severe bilateral cervical lymphadenopathy was present, with lymph nodes measuring approximately 2 cm, which were firm, elastic, and had limited mobility. There were no rashes, extremity changes, or hepatosplenomegaly.

Laboratory tests (Table 1) showed a white blood cell count of 18,600/μL and a C-reactive protein (CRP) level of 13.07 mg/dL, indicating systemic inflammation. Total bilirubin was elevated at 4.3 mg/dL.

**Table 1:** Laboratory findings on admission

Parameters	Value	Reference range
Complete blood count		
White-cell count (per μL)	18,600	4,000-10,700
Hemoglobin (g/dL)	12.7	11.5-14.4
Platelet count (per μL)	2,55,000	180,000-440,000
Coagulation tests		
Prothrombin time (PT)-International normalized ratio (INR)	1.41	0.90-1.15
Activated partial thromboplastin time (APTT) (sec)	33.7	26.9-38.1
Fibrinogen (mg/dL)	721	168-355
Serum chemistry		
Total protein (g/dL)	6.9	6.2-7.7
IgG (mg/dL)	1069	760-1670
Sodium (mmol/liter)	134	138-144
Potassium (mmol/liter)	3.3	3.6-4.7
Chloride (mmol/liter)	97	102-109
Urea nitrogen (mg/dL)	10.6	6.8-19.2
Creatinine (mg/dL)	0.64	0.42-0.80
C-reactive protein (mg/dL)	13.07	<0.3
Lactate dehydrogenase (U/liter)	201	124-222
Total bilirubin (mg/dL)	4.3	0.3-1.0
Alanine aminotransferase (U/liter)	17	13-30
Aspartate aminotransferase (U/liter)	8	Oct-42

**Figure 1:**
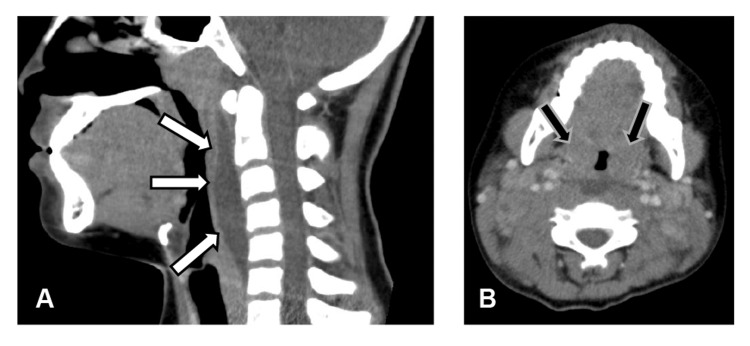
Contrast-enhanced CT scan of the neck of the patient (A) An irregularly shaped low-attenuation area (white arrow) was observed in the pharyngeal space, with no surrounding contrast enhancement. This finding is consistent with retropharyngeal edema, which can be seen in patients with Kawasaki disease. (B) Bilaterally enlarged cervical lymph nodes and hypertrophy of the palatine tonsils are observed (black arrows).

Neck contrast-enhanced computed tomography (Figure 1) revealed a hypodense area with poor contrast enhancement in the retropharyngeal space (Figure 1A), suggesting retropharyngeal edema rather than an abscess. Multiple enlarged cervical lymph nodes bilaterally and palatine tonsil hypertrophy (Figure 1B) were also observed.

Although KD was considered, the patient met only two principal criteria (fever and cervical lymphadenopathy). Given the concern for bacterial infection, empirical antibiotics were started.

On illness day 4, the patient developed lip erythema, meeting the diagnostic criteria for incomplete KD [2]. Specifically, the patient presented with four of the six principal clinical features of KD: persistent fever, bilateral conjunctival injection, lip erythema, and cervical lymphadenopathy. To ensure diagnostic clarity, we considered several differential diagnoses. Although fever and cervical lymphadenopathy raised suspicion for Epstein-Barr virus infection, it was considered unlikely due to the absence of hepatosplenomegaly and atypical lymphocytosis. High fever suggested the possibility of systemic juvenile idiopathic arthritis; however, the absence of arthritis and hepatosplenomegaly made this diagnosis less likely. In recent years, the similarities between KD and multisystem inflammatory syndrome in children (MIS-C) have been reported. However, MIS-C was ruled out in this patient due to the absence of a history of SARS-CoV-2 infection and a negative rapid antigen test for the virus performed on a nasopharyngeal swab at the time of admission. These considerations reinforce the diagnosis of KD. We initiated IVIG 100 g (2 g/kg), oral acetylsalicylic acid (ASA) 1500 mg (30 mg/kg), and intravenous methylprednisolone pulse therapy (IVMP) 1000 mg.

Following IVIG administration, serum IgG increased from 1069 mg/dL to 4193 mg/dL, and serum total protein concentration increased from 6.9 g/dL to 9.7 g/dL. The patient exhibited rapid symptom resolution, and prednisolone (PSL) 60 mg/day was initiated.

On the second day after IVIG administration, the patient experienced a recurrence of severe cervical pain, which was attributable to the accompanying cervical lymphadenopathy. As serum total protein levels exceeded 9.0 g/dL along with an increase in IgG levels, the risk of hyperviscosity syndrome was considered. To avoid excessive blood hyperviscosity, we administered a reduced IVIG dose of 50 g (1 g/kg) along with IVMP at 1000 mg on the following day. This approach was in line with the principles outlined in existing guidelines [2], which emphasize individualized treatment strategies for refractory cases. Following this treatment, serum IgG reached 3970 mg/dL, serum total protein was 9.1 g/dL, and both cervical lymphadenopathy and associated pain improved.

Two days after the second IVIG administration, the patient experienced a recurrence of symptoms, including bilateral conjunctival injection, erythema and dryness of the lips, and a mild elevation in body temperature from the 36°C range to the 37°C range. On the same day, IVIG was administered again at a dose of 1 g/kg. Due to the recurrent nature of the disease, we co-administered infliximab (IFX) 100 mg, a tumor necrosis factor-alpha inhibitor with a distinct mechanism of action from IVIG, based on evidence supporting its efficacy as a second-line therapy [6].

By illness day 15, serum IgG was 3642 mg/dL, serum total protein was 8.7 g/dL, and all symptoms except mild lip dryness resolved. Throughout the clinical course, no findings indicative of hyperviscosity syndrome, including visual impairment, diminished visual acuity, headache, dizziness, or manifestations of heart failure, were observed.

Table 2 presents the patient's serum IgG levels, total protein levels, hematocrit, and estimated whole blood viscosity (WBV) [7]. Prior to IVIG administration, WBV was within the reference range; however, a substantial increase was observed following IVIG administration.

**Table 2:** Changes in whole blood viscosity (WBV) over time WBV was calculated using the following formula, adapted from the literature [7]: WBV  = 0.12×HCT + 0.17(TP×10−2.07) where HCT represents hematocrit (%) and TP denotes serum total protein (g/dL). IVIG: intravenous immunoglobulin

Parameters	At admission	After the first IVIG (2 g/kg)	After the second IVIG (1 g/kg)	After the third IVIG (1 g/kg)	After discharge	Reference range
IgG (mg/dL)	1069	4193	3970	3642	1341	760-1670
Total protein (g/dL)	6.9	9.7	9.1	8.7	7.4	6.2-7.7
Hct (%)	38.4	35.2	42.5	39.2	39.8	36.0-46.0
WBV (208 sec^−1^)	15.9861	20.3621	20.2181	19.1421	17.0041	15.01 – 19.01

The patient was discharged on illness day 16 without any IVIG-related adverse effects or coronary artery lesions (CALs) (confirmed by serial echocardiography).

## Discussion

Kawasaki disease predominantly affects infants and young children, with few cases occurring in those over five years of age. Japanese epidemiological data indicate that among 12,966 KD patients, only 76 (0.59%) were aged 10 years or older [8]. However, as the study examined cases from 1997 to 1998, details regarding IVIG regimens were not available, and various dosing strategies were likely used during that period. In older children, diagnostic criteria are often met more slowly, potentially delaying IVIG initiation [9].

Intravenous immunoglobulin administration carries risks, including headache, chills, fever, fatigue, and facial flushing, which are generally mild. More severe complications, such as thromboembolism due to increased blood viscosity, heart failure from volume overload, aseptic meningitis, hemolytic anemia, and acute renal failure, have also been reported [4]. The increased body weight of older children necessitates higher IVIG doses, heightening the risk of these adverse effects.

A key challenge in the management of older children with KD is determining whether IVIG dosage can be reduced without compromising efficacy. Pediatric drug dosages are typically weight-based, but IVIG lacks a defined upper limit, resulting in proportional dose escalation with body weight. Studies have reported a higher incidence of IVIG-related adverse effects, including headache, vomiting, and dyspnea, in older children [10]. Though rare, severe complications such as hyperviscosity-related conditions cannot be disregarded. Notably, a case of cerebral infarction following IVIG administration has been reported in a patient with Kawasaki disease [11]. Similarly, cases of cerebral infarction occurring after IVIG have been reported in adults as well. In studies involving adult patients with dysimmune neuropathy, high-dose IVIG exceeding 35 g/day has been identified as a risk factor for thromboembolism [12]. Therefore, when the dose of immunoglobulin is increased, management of these side effects is considered important.

To optimize safety and efficacy, IVIG dose adjustments based on serum IgG levels have been proposed. Dalakas et al. measured blood viscosity before and after IVIG administration and reported that IVIG increased blood viscosity, which correlated with serum IgG levels [13]. Given that elevated blood viscosity can impair blood flow and potentially precipitate cardiovascular or cerebrovascular thromboembolic events, they emphasized the need for cautious IVIG administration with concurrent monitoring of blood viscosity. There is no established formula for estimating blood viscosity using serum IgG levels, nor a defined upper limit of serum IgG levels indicating a risk of hyperviscosity syndrome. However, a validated equation exists for estimating blood viscosity using hematocrit and serum total protein levels [7], which can be applied in clinical practice. Applying these reference ranges (Table 2) to the present case, WBV was within the normal range before IVIG administration but increased to the critically high range following IVIG administration. While these reference values are not specifically established for pediatric patients or Kawasaki disease, they provide useful insight into the changes in blood viscosity over time.

In the present case, IVIG 2 g/kg was administered on illness day 4, resulting in a serum IgG increase of 3124 mg/dL. By illness day 6, the patient experienced a recurrence of severe cervical pain, with serum total protein exceeding 9.0 g/dL, raising concerns about hyperviscosity syndrome. To mitigate this risk, a reduced IVIG dose of 1 g/kg was administered in combination with IVMP. Although the patient experienced another recurrence of symptoms, complete recovery was ultimately achieved without IVIG-related adverse effects or CALs following combination therapy with IVIG and IFX. Given that IVIG-induced hyperviscosity syndrome is a recognized risk, careful dose adjustment based on serum IgG and total protein levels is particularly important in older children with Kawasaki disease and in patients requiring repeated IVIG administration. Two previous studies targeting KD patients with average ages of 2.1 years and 2.4 years reported that, after the initial IVIG dose of 2 g/kg, serum IgG levels increased by approximately 2000 mg/dL [14, 15]. In this case, the increase in serum IgG levels following the initial IVIG greatly exceeded this value, suggesting that in older children with larger body size, IVIG administration at a dose of 2 g/kg may be associated with a greater increase in serum IgG levels. This raises the possibility that a 2 g/kg dose could be excessive from the perspective of preventing adverse effects.

## Conclusions

We encountered KD in a 13-year-old boy (53 kg) who required three IVIG infusions. Adjusting the IVIG dosage based on serum IgG levels effectively balanced the prevention of adverse events, such as hyperviscosity syndrome, while maintaining therapeutic efficacy in preventing the formation of coronary artery aneurysms. Future studies are needed to refine personalized IVIG dosing strategies for older children with KD.
